# Colorectal cancer screening barriers and facilitators among Jordanians: A cross-sectional study

**DOI:** 10.1016/j.pmedr.2023.102149

**Published:** 2023-02-13

**Authors:** Khaled Jadallah, Moawiah Khatatbeh, Tagleb Mazahreh, Aroob Sweidan, Razan Ghareeb, Aya Tawalbeh, Ansam Masaadeh, Bara Alzubi, Yousef Khader

**Affiliations:** a Department of Internal Medicine, King Abdullah University Hospital, Faculty of Medicine, Jordan University of Science and Technology, Irbid, Jordan; b Department of Basic Medical Sciences, Faculty of Medicine, Yarmouk University, Irbid, Jordan, and School of Health and Environmental Studies, Hamdan Bin Mohammed Smart University, Dubai, United Arab Emirates; c Department of Surgery, King Abdullah University Hospital, Faculty of Medicine, Jordan University of Science and Technology, Irbid, Jordan; d Department of Internal Medicine, Henry Ford Hospital, Detroit, MI, USA; e Department of Internal Medicine, Jordan University Hospital, Faculty of Medicine, University of Jordan, Amman, Jordan; f Department of Community Medicine, Public Health, and Family Medicine, Faculty of Medicine, Jordan University of Science and Technology, Irbid, Jordan

**Keywords:** CRC, Colorectal cancer, USPSTF, US preventive services task force, GPs, General practitioners, CI, Confidence interval, OR, Odds ratio, EMR, Electronic medical records, Colorectal cancer, Screening, Jordan, Barriers, Facilitators, Adherence, Attitudes, Perception

## Abstract

The factors affecting the adherence of Jordanians to colorectal cancer (CRC) screening remain underexplored. We examined the inhibitory and facilitating factors that influence the uptake of CRC screening among Jordanians. We conducted questionnaire interviews between April 2020 and June 2021 with 861 Jordanians aged 50–75. We analyzed the differences between proportions using the chi-square test. Binary logistic regression was conducted to determine factors associated with awareness of CRC and its screening. Of all participants, 41.7 % were aware of the necessity of screening for CRC, and 27.2 % were aware of at least one of the tests for CRC screening. However, only 17.2 % of participants underwent screening. In the multivariate analysis, participants with higher income (*p*-value < 0.001, odds ratio[OR] = 1.9, 95 % confidence interval [CI]: 1.4–2.7), higher level of education (*p*-value < 0.001, OR = 2.6, 95 % CI: 1.8–3.7), family history of colon cancer (*p*-value < 0.001, OR = 2.8, 95 % CI = 1.7–4.5), and those who had been screened for other cancers (*p*-value = 0.003, OR = 1.7, 95 % CI: 1.2–2.5) were more aware of the necessity of screening. Concerning barriers to screening, 'feeling well,' lack of physician endorsement, and difficult access to health care were the most commonly reported inhibitory factors (53.9 %, 52.3 %, and 31.9 %, respectively). The most commonly stated incentivizing factor was physician endorsement (82.3 %). Screening rates for CRC in eligible Jordanians remain low, albeit more than one-third of participants are aware of the necessity of screening. Enhanced awareness of barriers and incentivizing factors should help to prioritize national strategies to improve screening rates.

## Highlights

• Screening rates for colorectal cancer in eligible Jordanians remain low.
• The awareness of colorectal cancer is superior in participants with higher incomes.
• The most commonly reported reason for non-adherence to screening is “feeling well.”
• Physician endorsement is the most critical incentivizing factor for screening uptake.

## 1 Introduction

Colorectal cancer (CRC) is a significant public health problem worldwide. According to the GLOBOCAN 2020 estimates of cancer incidence and mortality, CRC is the third most common cancer, with 10.0 % of all new cases, and the second leading cause of cancer-related death, with 9.4 % of all cancer deaths (Sung et al., 2021). According to GLOBOCAN 2012, the highest CRC incidence in the Eastern Mediterranean region was found in Israel (36 per 100,000), followed by Jordan and Kazakhstan (26 and 23 per 100,000, respectively). The highest mortality rates were in Jordan, followed by Kazakhstan, Armenia, and Israel. In the Jordanian population, CRC is the most common cancer in men and the second most common in women, representing 18 % and 12.4 % of all cancers, respectively. The high mortality rate in Jordan is likely due to delayed diagnosis leading to a higher proportion of patients in an advanced stage of CRC. Hence, it is critical to implement CRC-controlling programs to increase screening rates, which have been shown to reduce CRC mortality (Gini et al., 2020).

Although CRC screening is strongly recommended in average-risk people, adherence rates remain low. The US Preventive Services Task Force (USPSTF) recommends screening for CRC starting at 50 years (Bibbins-Domingo et al., 2016). Rex et al. (2017) argued that screening for CRC should begin at 50 in average-risk persons, except in African Americans, whose limited evidence supports screening at 45 years. The healthcare authorities in Jordan endorse and finance CRC screening in agreement with the USPSTF recommendations. More recently, the guidelines from the USPSTF updated the task force recommendation for CRC screening to incorporate adults aged 45 to 49 years, which constitutes an important step in decreasing CRC morbidity and mortality (Davidson et al., 2021). A recent study from Jordan found that about 91 % of CRC cases were older than 45 years (Khatatbeh et al., 2018). Therefore, in agreement with the updated guidelines from the USPSTF and in light of our local data, the Jordanian Ministry of Health is considering including the age group 45–49 years in the CRC screening target.

A systematic review of eighteen studies on the impact of CRC screening on cancer-specific mortality in Europe revealed that screening significantly reduced mortality from CRC (Gini et al., 2020). Despite the conclusive evidence that screening significantly reduces CRC morbidity and mortality, this preventive health strategy is considerably underused among eligible individuals in developed and developing countries (von Wagner et al., 2011, Shapiro et al., 2012, Schreuders et al., 2015). Approximately-one-third of eligible adults in the United States did not undergo a screening procedure (Shapiro et al., 2012, Schreuders et al., 2015, James et al., 2002, Cokkinides et al., 2003), whereas lower rates of screening have been reported in several developing countries (Schreuders et al., 2015, Omran et al., 2015, Ravichandran et al., 2011).

Various barriers to screening have been reported in studies from different regions worldwide, including demographic factors, education, health insurance, income, knowledge about CRC and screening, patient and provider attitudes or structural barriers to screening [(Ravichandran et al., 2011, Gimeno García, 2012, Koo et al., 2010, Blumenthal et al., 2015). Factors affecting screening adherence can also be categorized into modifiable and non-modifiable factors involving patient, health care system, provider, and policy factors (Gimeno García, 2012).

In Jordan, a developing country, the proportion of eligible individuals who underwent screening is much lower than in developed countries (Omran et al., 2015). The factors associated with a low screening rate for CRC remain largely unknown.

The current study aimed to explore the attitudes, knowledge, and barriers concerning CRC screening among Jordanian adults and investigate the most critical incentivizing factors to improve screening uptake in eligible individuals.

## 2 Method

### 2.1 Setting and design

In this cross-sectional study, using a convenience sampling technique, we recruited participants aged 50–75 years from Jordan, a Middle Eastern country with approximately 10 million people. To ensure a representative sample of the general population, we recruited individuals from public spaces in rural, suburban, and urban areas in the country's Northern, Central, and Southern regions. In addition, patients and accompanying family members from outpatient clinics (both medical and surgical) in hospitals and healthcare centers in different parts of Jordan were sampled. The potential study subjects were approached and offered participation after a brief interview to confirm their eligibility to be included in the study. Recruitment took place between April 2020 and June 2021, with assistance from six medical residents trained to conduct semi-structured individual interviews. Interviews were approximately a 15-minute in duration.

### 2.2 Inclusion and exclusion criteria

Eligibility criteria for participation included self-identification as Jordanian, aged 50–75 years, and the provision of written informed consent before inclusion. Exclusion criteria were the previous diagnosis of CRC and the unwillingness to provide written consent. Additionally, patients attending the gastroenterology clinic for recto-colonic symptoms were excluded from the study.

### 2.3 Data collection tool

The study questionnaire was developed from similar surveys identified in the pertinent literature and adapted to the Jordanian context. The questionnaire comprised four parts: the first part was composed of questions on demographic and socioeconomic characteristics; the second part explored the knowledge and perceptions of CRC; the third part consisted of questions on the attitudes and barriers to CRC screening; the last part investigated potential facilitators of CRC screening uptake. The questionnaire underwent extensive review by two expert biostatisticians (MK; YK) for accuracy and face and content validity before pilot testing.

### 2.4 Ethical considerations

The study protocol was reviewed and approved by the Institutional Review Board at the Jordan University of Science and Technology (Grant No 20190170) and the participating hospitals and centers. Participants were granted a comprehensive explanation of the study protocol, emphasizing their right to refuse to participate. We obtained informed consent from all participants, and data confidentiality was sustained throughout. All study procedures were implemented following the Helsinki ethical declaration.

### 2.5 Statistical analysis

Data were analyzed using the statistical package for social sciences (SPSS) version 20. Data were described using means and percentages. The differences between proportions were analyzed using the chi-square test. We conducted a binary logistic regression to determine factors associated with awareness of CRC and its screening and characteristics associated with a history of screening for CRC. A *p*-value of<0.05 was considered statistically significant.

## 3 Results

### 3.1 Participants' characteristics

We approached a total of 921 individuals for the present study. Of those individuals, 212 were recruited from hospitals and 224 from health centers, whereas 180, 173, and 132 were from rural, suburban, and urban public spaces, respectively. Of the 921 individuals invited to participate, 861 (93 %) fulfilled the inclusion criteria. Table 1 illustrates the socio-demographic and clinical characteristics by gender.

**Table 1:** Socio-demographic and clinical characteristics of participants by gender.

**variable**	**Gender**			**Total**		***p*-value**	
	**Female**		**Male**				
	**n**	**%**	**n**	**%**	**N**	**%**	
**Age (year)**							0.298
45–55	156	35.7 %	159	37.5 %	315	36.6 %	
56–60	120	27.5 %	97	22.9 %	217	25.2 %	
>60	161	36.8 %	168	39.6 %	329	38.2 %	
**Income (JD)**							<0.001
≤400	266	64.3 %	159	37.9 %	425	51.0 %	
>400	148	35.7 %	261	62.1 %	409	49.0 %	
**Education level**							<0.001
<Bachelor	325	74.4 %	240	56.6 %	565	65.6 %	
≥Bachelor	112	25.6 %	184	43.4 %	296	34.4 %	
**Marital status**							0.004
Single	26	5.9 %	9	2.1 %	35	4.1 %	
Married/Ever married	411	94.1 %	415	97.9 %	826	95.9 %	
**History of medical illnesses**	247	56.5 %	243	57.3 %	490	56.9 %	0.869
**History of abdominal surgery**	175	40.0 %	90	21.2 %	265	30.8 %	<0.001
**Family history of colon cancer**	44	10.1 %	58	13.7 %	102	11.9 %	0.099
**Had screened for other cancers**	129	29.5 %	59	13.9 %	188	21.8 %	<0.001
**Having symptoms related to colonic disease**	139	31.8 %	134	31.6 %	273	31.7 %	0.949

The participants’ age (437 females and 424 males) ranged from 50 to 75 years, with a mean of 59.9 (SD ± 4.5) years. Females and males differed significantly in income, education, history of abdominal surgery, and screening for cancers other than CRC.

### 3.2 Awareness of CRC and its screening tests

The participants differed significantly in their awareness according to the studied characteristics. Table 2 shows the participants' awareness of CRC and its screening. Of all participants, 55.2 % were aware of CRC, 25.6 % were aware that CRC is the second most common cancer in Jordan, and 41.7 % were aware of the necessity of screening for CRC.

**Table 2:** The participants' awareness of CRC and its screening tests.

**Variable**	**Aware of CRC**			**Aware of CRC as second most common cancer**			**Aware of the necessity of screening for CRC**			**Aware of CRC screening tests**		
	n	%	*p*-value	n	%	*p*-value	n	%	*p*-value	n	%	*p*-value
**Gender**			0.073			<0.001			0.017			0.179
Female	228	52.2		107	24.5		165	37.8		110	25.2	
Male	247	58.3		113	26.7		194	45.8		124	29.2	
**Age (year)**			0.055			<0.001			0.002			0.211
≤55	180	57.1		101	32.1		151	47.9		96	30.5	
56–60	130	59.9		62	28.6		94	43.3		58	26.7	
>60	165	50.2		57	17.3		114	34.7		80	24.3	
**Income (JD)**			<0.001			<0.001			<0.001			<0.001
≤400	185	43.5		81	19.1		130	30.6				
>400	283	69.2		139	34.0		228	55.7		76	17.9	
**Education level**			<0.001			<0.001						<0.001
<Bachelor	256	45.3		96	17.0		190	64.2		105	18.6	
≥Bachelor	219	74.0		124	41.9			0.0		129	43.6	
**Marital status**			0.200			0.005			0.025			0.564
Single	23	65.7		16	45.7		21	60.0		11	31.4	
Married/Ever married	452	54.7		204	24.7		338	40.9		223	27.0	
**History of medical illnesses**			<0.001			0.107			0.002			0.008
no	236	63.6		105	28.3		177	47.7		118	31.8	
yes	239	48.8		115	23.5		182	37.1		116	23.7	
**History of abdominal surgery**			0.024			0.161			0.823			0.321
no	344	57.7		144	24.2		250	41.9		156	26.2	
yes	131	49.4		76	28.7		109	41.1		78	29.4	
**Family history of colon cancer**			<0.001			<0.001			<0.001			0.001
no	390	51.5		171	22.6		290	38.3		191	25.2	
yes	84	82.4		49	48.0		68	66.7		42	41.2	
**Had screened for other cancers**			0.124			<0.001			<0.001			0.003
no	362	53.8		142	21.1		256	38.0		167	24.8	
yes	113	60.1		78	41.5		103	54.8		67	35.6	
**Having symptoms related to colonic disease**			0.217			<0.001			<0.001			0.236
no	316	53.7		174	29.6		277	47.1		167	28.4	
yes	159	58.2		46	16.8		82	30.0		67	24.5	
CRC: Colorectal cancer												

### 3.3 History of screening for CRC

A total of 148 (17.2 %) participants reported that they underwent CRC screening using colonoscopy, flexible sigmoidoscopy, barium enema, CT colonography, guaiac-based fecal occult blood test, or fecal immunochemical test. Table 3 shows the history of screening for CRC according to the studied characteristics.

**Table 3:** Participants' history of screening colonoscopy or barium enema or other tests.

History of screening colonoscopy or barium enema or other tests					
	No		Yes		*p*-value
	n	%	n	%	
Female	368	84.2	69	15.8	
Male	345	81.4	79	18.6	
Age (year)					0.474
≤55	267	84.8	48	15.2	
56–60	179	82.5	38	17.5	
>60	267	81.2	62	18.8	
Income (JD)					<0.001
≤400	385	90.6	40	9.4	
>400	303	74.1	106	25.9	
Education level					<0.001
<Bachelor	500	88.5	65	11.5	
≥Bachelor	213	72.0	83	28.0	
Marital status					
Single	28	80.0	7	20.0	0.653
Married/Ever married	685	82.9	141	17.1	
History of medical illnesses					0.187
no	300	80.9	71	19.1	
yes	413	84.3	77	15.7	
History of abdominal surgery					0.025
no	505	84.7	91	15.3	
yes	208	78.5	57	21.5	
Family history of colon cancer					<0.001
no	643	84.8	115	15.2	
yes	70	68.6	32	31.4	
Had screened for other cancers					0.001
no	573	85.1	100	14.9	
yes	140	74.5	48	25.5	
Having symptoms related to colonic disease					0.170
no	494	84.0	94	16.0	
yes	219	80.2	54	19.8	

In the multivariate analysis (Table 4), participants with higher income, a higher level of education, a family history of colon cancer, and those having symptoms related to colonic disease were more likely to be aware of CRC.

**Table 4:** Multivariate analysis of factors associated with the participants' awareness of CRC and its screening tests.


Factor	**Aware of CRC**				**Aware of CRC screening tests**			
	OR	95 % CI		*p*	OR	95 % CI		*p*
**Having symptoms related to** **colonic disease** (yes. vs no)	1.6	1.1	2.2	0.006		-----		
**Income (JD)** (>400 vs ≤ 400)	1.9	1.4	2.7	0 < 0.001	2.1	1.4	3.0	0 < 0.001
**Education level** (≥Bachelor vs < Bachelor)	2.3	1.6	3.3	0 < 0.001	2.3	1.6	3.3	0 < 0.001
**History of medical illnesses** (yes. vs no)	0.7	0.5	0.9	0.010	-----			
**Had screened for other cancers** (yes. vs no)	-----				1.5	1.0	2.1	0.040
**Family history of colon cancer** (yes. vs no)	4.2	2.4	7.2	0 < 0.001	1.7	1.1	2.7	0.019
**History of abdominal surgery** (yes. vs no)	-----				1.5	1.0	2.1	0.035
CRC: Colorectal cancer								

Participants with university-level education, being screened for other cancers, having a family history of colon cancer, and those having a history of abdominal surgery had a doubled OR for being screened in the past. Results also found that participants whose income was > 400 JD were about three times more likely to have a history of CRC screening than those whose income was ≤ 400 JD. Table 5 illustrates these findings.

**Table 5:** Multivariate analysis of factors associated with a history of CRC* screening.

**Variable**	**OR**	**95 % CI**		***p*-value**
Having symptoms related to colonic disease (yes. vs no)	1.5	1.0	2.3	0.045
Income (JD) (>400 vs ≤ 400)	2.8	1.8	4.5	<0.001
Education level (≥Bachelor vs < Bachelor)	1.9	1.2	3.0	0.004
Had been screened for other cancers (yes. vs no)	1.9	1.2	2.8	0.004
Family history of colon cancer (yes. vs no)	2.0	1.2	3.3	0.006
History of abdominal surgery (yes. vs no)	1.9	1.2	2.8	0.003
* CRC: Colorectal cancer				

### 3.4 Barriers to CRC screening or reasons for not getting a CRC screening test

**Fig. 1:**
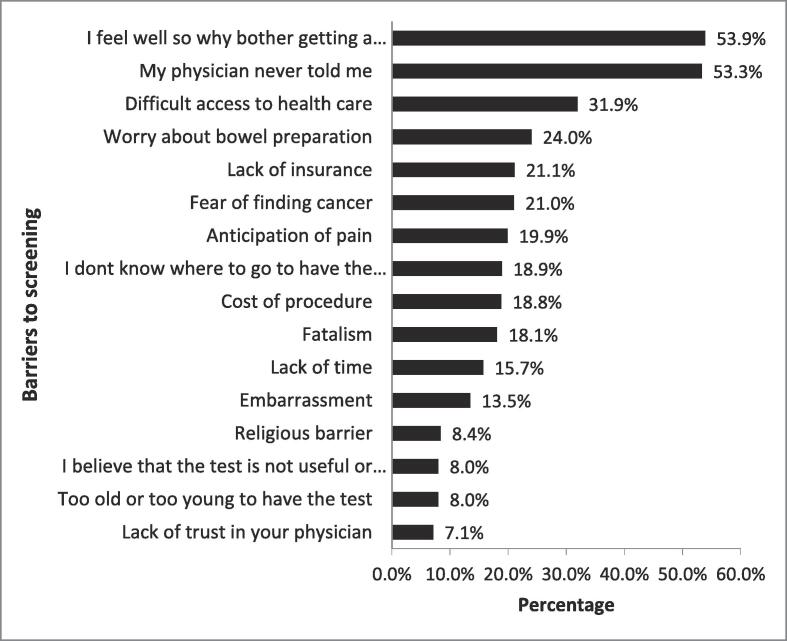
Barriers to adherence to colorectal cancer screening.

“Feeling well” was reported by more than half of participants (53.9 %) as a reason for not getting a CRC screening test, and “Never told by a physician to get screening” was another important reason. The third commonly reported reason was the difficulty accessing health care. Fig. 1 shows the barriers to or reasons for not getting a CRC screening test.

### 3.5 Incentivizing factors for getting CRC screening test

**Fig. 2:**
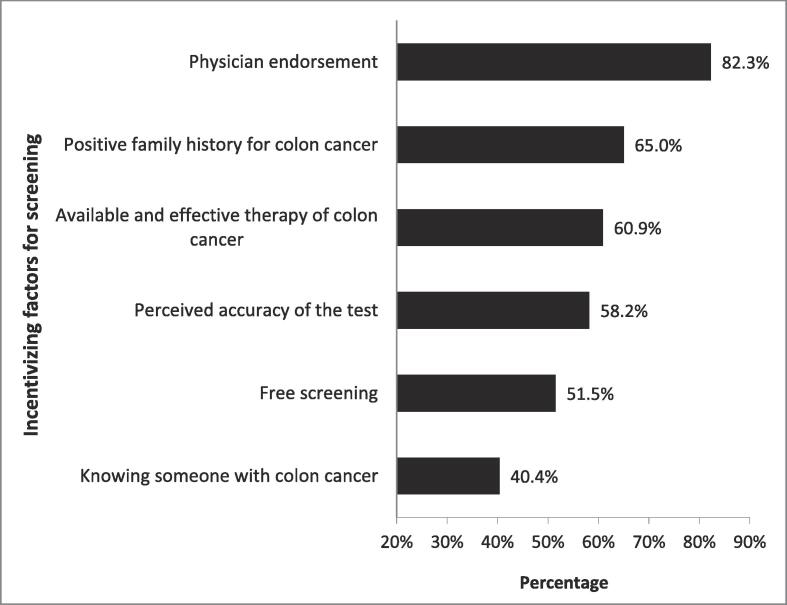
Incentivizing factors for getting a colorectal cancer screening test.

When asked about incentivizing factors for getting a CRC screening test (Fig. 2), >80 % of participants reported physician endorsement. About two-thirds considered a positive family history of colon cancer and available and effective therapy for colon cancer.

## 4 Discussion

Colorectal cancer (CRC) is one of the most commonly diagnosed cancers worldwide, with wide geographical variation in incidence and mortality (Sung et al., 2021, GLOBOCAN, 2012). Despite robust evidence that screening can decrease CRC incidence and mortality, only a tiny proportion of the target population worldwide adheres to CRC screening uptake (Gini et al., 2020, von Wagner et al., 2011, Shapiro et al., 2012, Schreuders et al., 2015).

This study provides in-depth insights into the main barriers to and facilitators of CRC screening uptake in screening-eligible Jordanians. The present study's most commonly reported factors for reluctance to adhere to CRC screening are “feeling well,” lack of physician endorsement, and difficult healthcare access. Conversely, the essential incentivizing elements of screening uptake were physician endorsement, a positive family history of CRC, and awareness of available and effective treatment for CRC. A recent study revealed similar themes about barriers to CRC screening among Latinos in Utah-USA and reported that they “did not know about CRC test”; “cost of CRC test”; and “has not had any CRC problems” (Warner et al., 2018).

Our results expand on findings from a previous cross-sectional study on the knowledge and beliefs of a sample of 160 Jordanians concerning CRC screening (Omran and Ismail, 2010). In that study, the data analysis revealed that most participants were not well informed about CRC and screening. Namely, 50 % (80 of the 160 participants) understood the seriousness of CRC, and the majority comprehended the benefits of CRC screening, whereas only one-third realized the barriers to CRC screening. In our study, more than half of the participants were aware of CRC, only one quarter was aware that CRC is the second most common cancer in Jordan, and 41.7 % were mindful of the necessity of screening for CRC. These results agree with a recent study from the USA, which reported that nearly half of the participants who were eligible for CRC screening had not heard of CRC (Warner et al., 2018). Moreover, low knowledge level was seen among the South Asian population in a qualitative study using focus groups (Ivey et al., 2018).

A systematic review with a *meta*-study synthesis of qualitative studies evaluating facilitators and barriers to uptake of CRC screening revealed that the decision to adhere to CRC screening depended on an individual's awareness of CRC screening (Honein-AbouHaidar et al, 2016). In our study, the understanding of CRC and the necessity of screening were relatively low (55.2 % and 41.7 %, respectively). Awareness affected views of cancer, attitudes towards CRC screening methods, and motivation for screening. The low awareness and other obstacles could explain the low screening rate (17.2 %) of eligible individuals in our population. This rate is far lower than reported in Western countries (Schreuders et al., 2015, Centers for Disease Control and Prevention (CDC), 2013, Holden et al., 2010). Therefore, the healthcare authorities in Jordan, despite the limited resources, should invest in public campaigns aimed at addressing misconceptions and various barriers to CRC screening.In Jordan, we perform colonoscopy opportunistically, unlike in many Asian and Western countries, where organized screening programs are in place (Schreuders et al., 2015, Levin et al., 2011). The low screening uptake rate in Jordanians is possibly due, at least in part, to the lack of organized screening programs. However, despite the approach to screening in the USA, like in Jordan, being largely opportunistic, screening rates are much higher (Miles et al., 2004). This discrepancy can be explained by differences in obstacles and incentivizing factors in those countries.

The vast majority of participants in the current study perceived at least two barriers to CRC screening uptake. This discrepancy between our data and the results reported by Omran et al. (2010) could be explained by the different methodologies used and the sample size of the two studies. In their systematic review of 77 articles, Wools et al. (2016) investigated the barriers and facilitators for CRC screening adherence. The authors found that females, younger age, low level of education, lower income, ethnic minorities, and single status were the most commonly stated barriers to CRC screening uptake.

A recent *meta*-analysis from the rural USA revealed that the most commonly reported obstacles for CRC were ‘ embarrassment or discomfort undergoing screening, lack of knowledge or perceived need for CRC screening, and lack of physician recommendation (Wang et al., 2019). Furthermore, that study revealed that healthcare provider features, such as health insurance and a usual source of care, were also frequently reported barriers to CRC screening uptake. In a population-based Canadian study of predictors of non-adherence to CRC screening among immigrants, Middle Eastern and North Africans (Arabs) were the third most non-adherent (39.7 %) group of immigrants [(Shen et al., 2018). Cultural values and beliefs may explain such findings. A South Asian study corroborated this suggestion, reporting that sentiments of shame and modesty might prevent CRC screening [(Warner et al., 2018). The results of that study agree with those of a study from Jordan [(Omran et al., 2015).

In the present study, the most relevant facilitators of screening uptake were physician endorsement, positive family history of CRC, and awareness of available and effective treatment for CRC. Our results partially agree with other studies investigating inhibitory and incentivizing factors for screening (Omran et al., 2015, Ivey et al., 2018, Honein-AbouHaidar et al., 2016, Wools et al., 2016, Wang et al., 2019, Shen et al., 2018). Several investigators have reported physician endorsement, especially general practitioners (GPs), as among the most important facilitators of screening uptake, regardless of the recommended screening method (Raine et al., 2016, Hewitson et al., 2011). A British study found that adding a statement of GPs endorsement to the standard “bowel cancer screening program invitation letter” increased the chances of participation in the screening program by 7 % with no significant upfront cost (Hewitson et al., 2011). A cross-sectional, primary care-based study from Malaysia aimed to determine the intention and the uptake of CRC screening and to explore the related motivators and barriers after raising awareness with brief health education (Chan et al., 2021). In that study, the authors found that physicians-provided health education is more effective than a standardized education session in promoting adherence to CRC screening. In Jordan, the primary care health system depends mainly on GPs. Earlier studies (unpublished data) showed that more than half of the Jordanian GPs do not routinely recommend CRC screening for eligible individuals. Thus, we believe that fostering the attitudes of GPs toward CRC screening is one of the most critical interventions to affect CRC screening uptake positively. Therefore, the healthcare authorities should emphasize the role of GPs in enhancing CRC screening rates. A recent systematic review investigated the facilitators and barriers to implementing interventions to enhance CRC screening uptake in primary care practice (Adhikari et al., 2022). In that study, the authors found that engagement of the clinic team, leadership team, and partners were the most critical implementation facilitators. Other significant facilitators were clinics’ motivation to improve CRC screening rates, use of the electronic medical records (EMR) system, continuous monitoring and feedback system, and having a helpful environment for implementation. Conversely, time constraints for the clinic team to dedicate to a new project, challenges in getting accurate, timely data related to CRC screening, little ability or support to use the EMR system, and disconnection between clinic team members were the most commonly reported implementation barriers. Therefore, we believe in the joint effort of researchers, decision-makers, primary care physicians, and program developers in designing action plans and developing strategies to optimize implementation. Furthermore, to increase public awareness of the CRC, there is a need for continuous public health campaigns.

The current study adds to the existing body of knowledge by assessing the most significant number of factors of non-adherence to CRC screening among a large and representative sample of eligible Jordanians. Additionally, collecting data via face-to-face interviews rather than self-reported questionnaires substantiated our results. On the other hand, a limitation of this study was that we did not examine our data in light of different screening methods. In contrast, previous work has identified different screening patterns by the modality of a screening exam (Shapiro et al., 2012). Various forms of CRC screening should be examined as outcomes in future studies, especially as specific modalities are recommended over others.

## 5 Conclusions and future directions

Screening rates for CRC in eligible Jordanians remain very low, albeit>40 % of participants are aware of the necessity of screening. Healthcare authorities must optimize CRC screening to reach the ideal target of reducing the incidence of the disease and, eventually, its mortality. Awareness of barriers and incentivizing factors should help prioritize national strategies to increase screening rates. Additionally, we should not ignore that most screening, especially in low and middle-income countries, is performed opportunistically with no concrete structure.

We expect the present study's findings to positively affect policymakers, healthcare providers, and professional organizations in Jordan about CRC screening. Future studies using qualitative research methods targeting stakeholders, including policymakers, physicians, and community members, should investigate the barriers and facilitators of the full spectrum of available CRC screening options.

Ethics approval and consent to participate.

The study protocol was approved by the Institutional Review Board at King Abdullah University Hospital and the Committee on Human Research at the Jordan University of Science and Technology (Grant No 2019/0170), and the participating hospitals and centers. We obtained written informed consent from all participants**.**

Consent for publication.

Not applicable.

Availability of data and materials.

The datasets used and analyzed during the current study are available from the corresponding author on reasonable request.

Funding.

This work was financially supported by the Deanship of Scientific Research at the Jordan University of Science and Technology (Grant No 2019/0170).

## Declaration of Competing Interest

The authors declare that they have no known competing financial interests or personal relationships that could have appeared to influence the work reported in this paper.

## Data Availability

Data will be made available on request.
